# Lipopolysaccharide-binding protein expression is associated to the metastatic status of osteosarcoma patients

**DOI:** 10.1016/j.jbo.2022.100451

**Published:** 2022-08-04

**Authors:** Clément J.F. Heymann, Christine Bobin-Dubigeon, Javier Muñoz-Garcia, Denis Cochonneau, Emilie Ollivier, Marie-Françoise Heymann, Dominique Heymann

**Affiliations:** a University of Amsterdam, Academic Medical Center, Swammerdam Institute for Life Sciences, Amsterdam, the Netherlands; b Institut de Cancérologie de l’Ouest, Tumour Heterogeneity and Precison Medicine Laboratory, Saint-Herblain, France; c Nantes Université, CNRS, UMR6286, US2B, Nantes, France; d Institut de Cancérologie de l’Ouest, Research Pathology Platform, Saint-Herblain, France; e University of Sheffield, Department of Oncology and Metabolism, Medical School, Sheffield, UK

**Keywords:** Osteosarcoma, Intratumour bacteria, Gram-negative microbiota, Tumour-associated macrophages, LPS, Lipopolysaccharide, LBP, LPS-binding protein, MTP-PE, Muramyl tripeptide-phosphatidyl ethanolamine, OS, Osteosarcoma, TLR4, Toll-like receptor 4

## Abstract

Osteosarcoma (OS) is a rare malignant primary bone tumours characterized by a high genetic and cell composition heterogeneity. Unfortunately, despite the use of drug combinations and the recent development of immunotherapies, the overall survival has not improved in the last four decades. Due to the key role of the tumour microenvironment in the pathogenesis of OS, a better understanding of its microenvironment is mandatory to develop new therapeutic approaches. From retrospective biological cohorts of OS, we analysed by immunohistochemistry the presence of lipopolysaccharide (LPS)-binding protein (LBP) in diagnostic biopsies with local disease and compared their level of infiltration to patients suffering from metastatic status. LBP is considered as a marker of LPS exposure and can indirectly reflect the presence of Gram-negative microbiota. LBP were detected in the cytoplasm of OS cells as well as in tumour-associated macrophage. Tumour samples of patients with local disease were significantly enriched in LBP compared to tumour tissues of patients with metastatic status. Lung metastatic tissues showed similar level of LBP compared to paired primary tumours. Overall, this study strongly suggests the presence of Gram-negative bacteria in OS tissues and demonstrated their significant differential level according the metastatic status. This tumour-associated microbiome may help in the conceptualisation of new therapeutic approach to trigger efficient therapeutic responses against cancer.

## Highlights

• Intratumour Gram^-^ bacteria can be detected in OS.
• Enriched intratumour in Gram^-^ bacteria infiltrate is associated with local disease.
• A poor Gram^-^ bacteria infiltration may be predict a higher risk of metastasis.
• Immune stimulation of OS by LPS represents a potential therapeutic option.

## 1 Introduction

Cancer defines a large group of neoplastic diseases, induced by both intrinsic and extrinsic causative factors. Uncontrolled growth and spread of malignant cells lead to the formation of tumours characterised by specific genomic, molecular and phenotypic profiles. Tumours show a large diversity of ecosystem composed of extracellular matrix, blood vessels, and of various immune cells (e.g. T lymphocytes, macrophages), acting cooperatively with stromal cells toward the active progression of cancer [1]. This tumour ecosystem has a strong influence on cancer cell proliferation/apoptosis/migration, tissue vascularisation, immune response and also contributes to the control of drug sensitivity or resistance [2], [3]. The recent characterisation of intracellular bacteria in various tumours led to decipher the functional relationship between tumour microenvironment and cancer onset. Nejman *et al.* were the first to identify intratumour bacteria in both immune and cancer cells, including bone tumours [4]. Tumour microbiome increases the complexity of the already sophisticated cancer ecosystem. A better understanding of the role played by intratumour bacteria *in vivo* would help to the therapeutic decision and allow a better patient stratification [5].

Tumours possess all the required properties to support high bacterial prevalence. In complement to the natural ability of cancer cells to hide from host immune surveillance, the abundant vasculature surrounding tumours make them perfect lying spots for circulating bacteria [6], [7]. Likewise, tumour necrotic regions also release high levels of nutrients (e.g. purines) and chemoattractant compounds (e.g. aspartate), which emphasize survival and propagation of bacteria in cancer cells. Overall, the tumour microenvironment can form an immune tolerant region, where bacteria can escape host immune defenses and proliferate at ease [6]. Although convinced of their existence, uncertainties concerning the origin and functions of intratumour bacteria remain. Based on the recent studies, tumour microbiome is intrinsically heterogeneous with different bacterial communities, specific of each tumour type. Furthermore, the comparison of the microbiome between tumour and related healthy tissues confirmed the complete diversion of tumour microbiomes from standard organ-derived microbial profiles [4]. However, the study of intratumour microbiota remains very challenging. Indeed, the microbial biomass of tumors is highly limited. There is no consensus on the methodological approaches that can be used for bacterial detection and the source of tumour tissues (e.g. Formalin-Fixed Paraffin-Embedded samples) can also restrict the analytical process. In addition, risks of extrinsic contamination during the analysis process cannot be ignored, which adds an unpredictable factor to the entire characterisation process.

Osteosarcoma (OS) is a rare oncological entity that belongs to the mesenchymal tumour family [8]. Unfortunately, the 5-year survival rate of OS patients has not been improved in the last four decades with a rate around 60 % for patients with no clinically detectable metastasis and 30 % for patients with metastatic foci detectable at the time of diagnosis [8]. Regardless of the nature of cancer entities, the tumour microenvironment plays a crucial role in the overall pathogenesis of OS [9], [10], [11]. The present study aimed to compare the LBP expression as a biomarker of Gram-negative bacteria exposure in three OS biological cohorts composed by primary tumour tissues of patients with and without metastatic status.

## 2 Materials and methods

### 2.1 Patients

159 OS cases were retrospectively included in the present study. The experimental procedures were carried out in accordance with both the ethical standards of the responsible institutional committee on human experimentation, and with the Helsinki Declaration (Authorisation: French Research Ministry n° 2008-402). From all these cases, 36 and 123 patients were diagnosed with (OS Meta^+^) and without (OS meta^-^) metastatic disease, respectively. Out of these 159 individuals, 22 patients (16 OS meta^-^, 6 OS Meta^+^), for whom no pre-chemotherapy sample was available, were excluded from the analysis. 13 additional patients (11 OS meta^-^, 2 OS Meta^+^) for which the diagnosis changed after the surgical resection of the sample, were also removed from the study. After the pre-selection process, 50 patients out of 159 were conserved for the study, including 22 patients defined as OS meta^-^ and 28 patients as OS Meta^+^ (Table 1) [12]. The median age was similar between patients with and without metastatic diseases [mean: 23.1 year old (total population), 22.5 year old (OS meta^-^), 23.7 year old (OS Meta^+^)]. The majority of tumors were observed in femur (56 %) and lung was the main metastatic site in 75 % of OS Meta^+^ (Table 1) [12].

**Table 1:** Clinical profile of the osteosarcoma cohort.

Number of patients	50
Gender (%)	Female (40) / Male (60)
Age (mean, year)	23.1 (min. 7- max. 80)
BMI (kg/m^2^)	25.6
Primary tumour site, n (%) Femur Tibia Humerus Ulna Others	28 (56)11 (22)8 (16)1 (2)2 (4)
Metastatic status (mean, %)	* OS Meta^+^ (56)
Tumor size (mean, cm)	* OS meta^-^ (9.5) OS Meta^+^ (11.2)

*OS Meta^+^: osteosarcoma patients with metastatic foci clinically detectable; OS meta^-^: osteosarcoma patients with local disease.

### 2.2 Tissue preparation and immunohistochemistry

#### 2.2.1 Tissue microarray preparation and histological analysis

OS tissue samples (primary tumours and lung metastatic foci) were formalin-fixed (10 %), decalcified with nitric acid or by electrolysis with SAKURA TDE^TM^ 30 (Japan) and paraffin-embedded. Diagnosed was carried out according to the World Health Organization classification of malignant bone tumours by two independent pathologists [13]. From this tumour samples, tissue microarrays were prepared. Three core samples of 1 mm in diameter were performed per osteosarcoma sample, in the most representative areas of the HES sections, and then embedded in paraffin blocks. 3 µm sections were used for immunohistochemistry investigations.

#### 2.2.2 Immunohistochemistry

After dewaxing and rehydration of 3 μm thickness sections of formalin fixed paraffin embedded samples, antigen retrieval was carried out at 60 °C for 20 h in acidic antigen retrieval solution at pH 6. To maximise staining repeatability, immunohistochemistry was carried out using the BOND-III/BOND RX IHC automatic stainer (Leica, Biosystem, France). After retrieval of antigen epitopes, endogenous peroxidase was blocked with 3 % H_2_O_2_ solution. anti-lipopolysaccharide (LPS)-binding protein monoclonal antibody (anti-LBP, Ref. 863801, dilution: 1/100, BioLegend, USA) was mixed with specific diluent (Leica Bond, ref. AR9352). Immunohistochemical staining procedure was completed with a specific HRP-polymer left and DAB revelation labeling before final counter-staining by hematoxylin. The preliminary optimization of immunohistochemical parameters was manually performed from human colon tissues (Centre de Ressources Biologiques-Tumorothèque ICO, Saint-Herblain, FR, authorization N° DC- 2018-3321) [14]. Immunohistochemistry was scored by independent pathologists, according to the levels of staining detected from each cell. A semi-quantifying approach based on the intensity the immunoreactivity was then used and defined as negative, weak (1+), average (2+), or high (3+) [15].

## 3 Statistical analysis

Statistical analyses were performed using R software version 4.1.2. All results were compared, using a one-way analysis of variance (ANOVA) followed by a Tukey post-hoc analytic test. P-value ≤ 0.05 was considered statistically significant.

## 4 Results

### 4.1 Osteosarcoma cells and tumour-associated macrophages express LBP

**Fig. 1:**
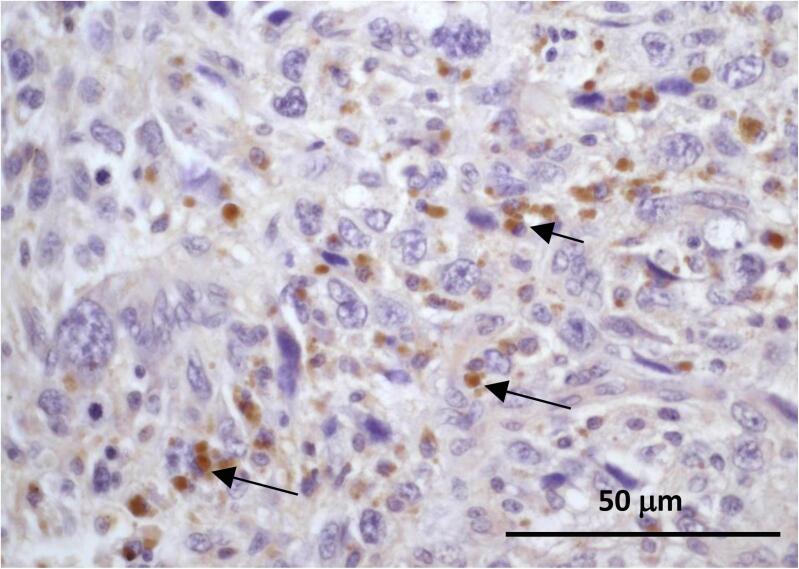
**LPB Positive immunoreactivity of OS cells as a reflect of LPS exposure and potential presence of Gram-negative microbiota in the tumour ecosystem.** Following preliminary antigen retrieval, 3 μm OS section was incubated with anti-LBP primary antibody (1/100) at 37 °C for 1 h. HRP staining was then performed, followed by hematoxylin counterstaining. Positive anti-LBP immunostaining was observed within cancer cytoplasm, suggesting the presence of bacterial LPS (from Gram-negative bacteria) inside OS cells (arrow). Original magnification: X400.

**Fig. 2:**
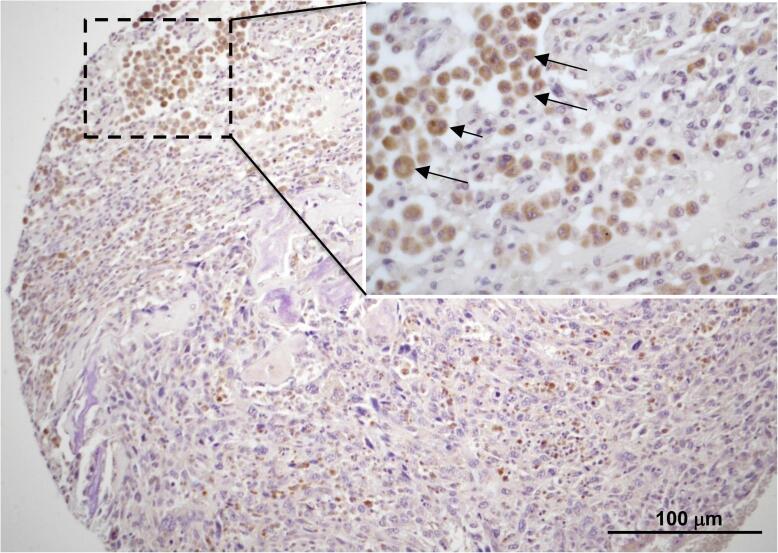
**Detection of bacterial LPS in tumour associated macrophages.** LPS were detected indirectly by immunohistochemistry revealing the presence of LBP. Positive immunostaining was observed in the cytoplasm of host phagocytic cells surrounding the tumour (arrow). Original magnification: X100, X400 (insert).

LBP binds to LPS which is the major wall component of all Gram-negative symbiotic and pathogenic bacteria and initiates the immune response in infectious situations. The detection of LBP at the tissue level can be used of an indirect biomarker of symbiotic and/or pathogenic Gram-negative bacteria or/and LPS exposure [4], [16]. We then assessed the LPB expression by immunohistochemistry to indirectly determine the prevalence of Gram-negative bacteria in tissue samples of OS. Anti-LPB immunostaining was identified in the tumor microenvironment of 21 out of 50 OS primary sites. As shown in Fig. 1, positive immunostaining was predominantly observed in the cytoplasm of cancer cells suggesting an intracellular prevalence of Gram-negative microbiota exposure in OS tissues. In addition to the positivity of OS cells, LBP was detected in tumour-associated macrophages (Fig. 2).

### 4.2 LBP is preferentially associated in osteosarcoma patients with local disease

In order to better determined the biological value of LBP in the pathogenesis of OS, we semi-quantified the LBP expression as biomarker of the microbiota infiltrate in primary tumour samples (diagnosed biopsies) of OS patients with and without metastases and in the corresponding lung metastatic foci. Biopsies collected from patients with local disease showed 77 % of positive anti-LBP immunoreactivity (Table 2) contrasting to patients with metastatic disease who had only 39 % of biopsies positive to LBP (Table 3). In addition to this significantly higher positivity (*p* < 0.001), the intensity of anti-LBP staining was also increased in OS meta^-^ group [1^+^: 36 %; 2^+^: 18 %; 3^+^ 23 % with 26,3% of positive cells] (Table 2). In OS Meta^+^ group, only 15 % of cancer cells were stained including 32 % with 1^+^ intensity, 4 % with 2^+^ and no stained cells with 3^+^ level (Table 3).

**Table 2:** Anti-LBP immunostaining of osteosarcoma samples from patients with no metastatic foci clinically detectable.

Patient reference	LBP IHC intensity	% of positive tumour cells
1	1+	20
2	3+	57
3	2+	25
4	3+	60
5	3+	60
6	*	*
7	2+	20
8	1+	20
9	3+	53
10	*	*
11	2+	80
12	2+	40
13	1+	10
14	3+	40
15	Negative	
16	1+	5
17	Negative	
18	Negative	
19	Negative	
20	Negative	
21	1+	25
22	1+	10

IHC: immunohistochemistry; *samples not analysable.

**Table 3:** Anti-LBP immunostaining of osteosarcoma samples from patients with metastatic foci clinically detectable.

	Primary tumour		Metastatic foci	
Patient reference	LBP IHC intensity	% of positive tumour cells	LBP IHC intensity	% of positive tumour cells
23	Negative		*	*
24	1+	15	*	*
25	Negative		Negative	
26	Negative		Negative	
27	1+	10	Negative	
28	Negative		1+	30
29	1+	15	Negative	
30	Negative		1+	40
31	Negative		Negative	
32	Negative		Negative	
33	Negative		Negative	
34	Negative		Negative	
35	Negative		Negative	
36	Negative		1+	15
37	1+	20	*	*
38	Negative		1+	10
39	Negative		*	*
40	Negative		1+	10
41	1+	15	1+	5
42	Negative		1+	10
43	Negative		*	*
44	1+	30	1+	5
45	1+	20	*	*
46	1+	10	1+	5
47	1+	10	2+	20
48	1+	10	*	*
49	1+	15	Negative	
50	Negative		Negative	

IHC: immunohistochemistry; *samples not analysable.

We then compared the LBP expression between primary tumours and paired metastastic foci. As summarised in Table 3, 64 % of lung metastastic nodules did not show any significant immunoreactivity against LPB, 32 % were characterised by 1^+^ intensity and 4 % by 2^+^. The mean value of LBP-positive cancer cells was 16.6 %. Four negative primary biopsies were associated with moderate LBP immunostaining (1^+^) of lung metastases (Patients ref. 28, 36, 38, 42).

## 5 Discussion

Despite the emergence of new diagnostic tools, cancer remains one of the principal causes of death worldwide. The number of cancer cases is dramatically rising, simultaneously to the increase of life expectancy in developed countries and cancer is related to the diseases of old age. Each tumor is characterized by specific and pleomorphic histological/molecular features making cancer a highly heterogenous pathology [17]. OS exhibit similar criteria with high heterogeneous profiles not only in term of genetic properties but also in cellular components [9], [10], [11], [18]. Identified in tumours hundred years ago, live-pathogenic bacteria have thenceforth been an active part of therapeutic development in cancers. Over the last five years, several research teams have revealed and identified the presence of non-pathogenic microbiota in the ecosystem of the most common solid tumors [4], [19], [20], [21], [22]. The present study revealed the LBP expression in OS tissues and highlights their potential contribution to the tumour microenvironment [4]. Positive staining was also noticed in host phagocytic cells, surrounding most tumour tissues. Interestingly, biological cohorts analysed displayed differential LBP staining results according to the metastatic status of OS patients. Indeed, in contrast to diagnosed biopsies of patients suffering from metastases characterised by a moderate LBP immunoreactivity, primary tumour biopsies of patients with a local disease showed high percentage of positive cancer cells including average and high immunoreactivity. Lung metastatic foci showed similar pattern of LBP immunostaining compared to the paired primary tumours.

Macrophages strongly contribute to the immune infiltrate in OS tissue and pre-clinical and clinical studies demonstrated the therapeutic interest of their activation [10], [12], [23]. Muramyl tripeptide-phosphatidyl ethanolamine (MTP-PE) is an analogue of muramyl dipeptide, a substance contained within the cell wall of Gram-positive and Gram-negative bacteria which acts as macrophage-activating agents [24], [25], [26]. Patients suffering from localised OS treated by liposomal-MTP-PE may significantly improve their overall survival by the promotion of M1-macrophage [27], [28]. In 2016, we characterised the immune infiltrate in OS by using the same biological cohorts described in the present manuscript [12]. We observed a marked infiltration of M1-polarised macrophages characterised by INOS immunoreactivity in local disease compared to OS patients with diagnosed metastases. The anti-tumour properties of M1-macrophages have been hypothesised from pre-clinical observations [26], [29]. MPT-PE activated M1-macrophages inhibit OS growth and this anti-tumour activity was partly associated with the release of TNF-α and IL1-β [26]. In addition, the long-term follow up of tumour growth has been associated to a switch of macrophage polarisation from M1 to M2 phenotype and to the therapeutic escape [29]. Interestingly, in the current study, the LBP expression and the number of M1-polarised macrophages were similarly increased in OS meta^-^ group compared to OS Meta^+^ group suggesting a potential functional relationship between intra-tumour microbiota and the local immune surveillance by macrophages (Table 2, Table 3, [12]).

LPS also called bacterial endotoxins are members of a class of phospholipids found in the outer membrane of Gram-negative bacteria. Toll-like receptor 4 (TLR4) binds LPS then mediating inflammatory responses and controlling innate immunity [30], [31]. In the present study, LBP detected in the cytoplasm of cancer cells, was used as an indirect biomarker of LPS exposure and consequently of Gram-negative bacteria in the tumour ecosystem [16]. Indeed, LBP is an acute phase protein that binds to LPS leading to the induction of immune responses by presenting LPS to immune cell by CD14 and TLR4. A recent gene set enrichment analysis showed that immune-related pathways were enriched in the low-risk group of OS patients [32]. This study confirmed that both M1 and M2 polarised macrophage markers were overexpressed in local disease and macrophages negatively correlated with the risk score [33]. TLR4 expression appeared lower in tumour tissues than in peritumoral area and its expression at the cell membrane is tightly controlled and, in particular, TLR4 is internalised after the LPS binding [33]. In addition, these authors identified a risk score based on three immune-related genes including TLR4. Overall, these data are in agreement with our observation, Gram-negative bacteria may influence OS progression by controlling macrophage activation and TLR4 trafficking.

TLR4 agonists have been recently developed to increase the therapeutic efficacy of immune checkpoints inhibitors [34], [35]. A recent phase 1/2 clinical trial assessed the therapeutic benefit to combine a TLR4 agonist (G100) with pembrolizumab in follicular lymphoma. G100 intratumour injection was not associated with any toxicity and resulted in overall response rate of around 30 % and an interesting abscopal effect in more than 70 % of patients [36]. This study demonstrates the therapeutic interest to use TLR4 agonists to produce immune-mediated antitumor response for tumours associated with TLR4 activation. Clinical observation is in favour of TLR4-based therapeutic option in osteosarcoma. In a large cohort of OS patients that enrolled more than 400 patients, Jeys *et al* observed a better survival at 10 years in patients who developed an infection within one year of orthopaedic surgery (84.5 % in the infected group vs 62.3 % in the non-infected group) [37]. Activation of TLR4 by LPS administration in OS bearing mice strengthened the clinical observation [38]. Indeed, LPS increased CD8^+^ T lymphocyte infiltration into lung metastatic foci and concomitantly reduced OS progression. Intra-tumour microbiota observed in OS may contribute to control the tumour development and consequently TLR4 activation by specific agonists may represent a potential therapeutic opportunity to stimulate the immune response against cancer cells. Interestingly, a recent study has shown superior overall survival in patients suffering from clear cell renal cell carcinoma who have not been treated with antibiotics [39]. This observation strengthens the potential contribution of intratumour microbiome in the local immunity and tumour progression.

This report argues in favor of intratumour bacteria associated to bone sarcomas [4] and shows a significant differential expression of LBP in OS tissues discriminating local from metastatic disease. Such Gram-negative bacteria may contribute to the tumour ecosystem in absence of active infection. These bacteria are not proliferating and must be considered as symbiotic partners which may participate in the control of the local immunity [4], [7]. Their non-proliferative property makes difficult their detection. Further investigations will be mandatory to detect and characterise in large prospective series of OS the presence of intratumour bacteria. The intratumour microbiome could be studied at the genetic/molecular levels that was technically not possible from retrospective decalcified and paraffin embedded samples as used in the present work. The first investigational option is the 16S ribosomal RNA sequencing which allows for the identification and clustering of bacteria into distinct taxonomic groups [40]. The shotgun-based approaches recovering all genome sequences may be the second option to profile taxonomic composition and functional properties of the intratumoural microbiome [41]. The third option could evaluate the effects of intratumoural administration of purified Gram-negative bacteria wall proteins upon host immune responses by using animal models as it has been used for MTP-PE studies [24], [25], [26].

The correlation of gram-negative bacterial content with the immune infiltrate may allow the development of new immunomodulatory drugs in OS. Such prospective study should be associated with a controlled aseptic procedure of tissue collection and manipulation. However, even if the samples studied in the present report were collected in the respect of the conventional pathological procedure with a risk of cross contamination, all patients enrolled in our study were treated by the same surgery team and procedure, all tissues were processed, conserved similarly by the same team of pathologists and were analysed using the same protocol. Despite all its pitfalls, a marked significant intratumour level of LBP expression reflecting LPS exposure was clearly identified strengthening the value of our biological observation.

Intratumour microbiome is an emerging topic that should be considered as a part of the tumour ecosystem that may contribute to the control of immune associated tumour progression. In addition to their prognostic value, intratumour microbiome and more particularly LPS constitute an interesting therapeutic option that should be evaluated further. Complementary *in-vitro* and *in-vivo* studies are now required to dig into the precise characterisation of OS microbiome that may pave the way of new therapeutic development.

## Declaration of Competing Interest

The authors declare that they have no known competing financial interests or personal relationships that could have appeared to influence the work reported in this paper.
